# Assessing the causal effects of *Eubacterium* and *Rumphococcus* on constipation: a Mendelian randomized study

**DOI:** 10.3389/fmicb.2024.1376232

**Published:** 2024-07-31

**Authors:** Xiao Zhang, Jiang Chen, Feng He, Wenchun Du, Xianhao Yu

**Affiliations:** ^1^Guizhou University of Traditional Chinese Medicine, Department of Colorectal Medicine, Guiyang, Guizhou, China; ^2^Guizhou University of Commerce, Computer and Information Engineering College, Guiyang, China

**Keywords:** constipation, Mendelian randomization, causal relationship, GWAS, *Rumphococcus*, *Eubacterium genus*

## Abstract

**Background:**

Constipation is affected by a number of risk variables, including cardiovascular disease and growth factors. However, the impacts of gut flora on constipation incidence has not been shown. This work, Single-Variable Mendelian Randomization (SVMR) was utilized to estimate the causal relationship between the *Eubacterium genus* or *Rumphococcus*, and constipation.

**Methods:**

Data for constipation, *Eubacterium genus* and *Rumphococcus* were taken from the Integrated Epidemiology Unit (IEU) open GWAS database. Including 218,792 constipation samples, and there were 16,380,466 Single Nucleotide Polymorphisms (SNPs) for constipation. The ids of *Eubacterium genus* and *Rumphococcus* were sourced from MiBioGen database. The sample count for the *Eubacterium genus* was 17,380, with 656 SNPs. In addition, the sample size for *Rumphococcus* was 15,339, with 545 SNPs. The SVMR was performed to assess the risk of *Eubacterium genus* and *Rumphococcus* in constipation using weighted median, MR Egger, simple mode, inverse variance weighted (IVW), and weighted mode. Finally, we did a sensitivity analysis that included a heterogeneity, horizontal pleiotropy, and Leave-One-Out (LOO) test to examine the viability of the MR data.

**Results:**

The SVMR revealed that the *Eubacterium genus* and *Rumphococcus* were causally connected to constipation, with *Rumphococcus* (*P* = 0.042, OR = 1.074) as a hazardous factor and *Eubacterium genus* (*P* = 0.004, OR = 0.909) as a safety factor. Sensitivity tests then revealed the absence of variability between the constipation and the exposure factors (*Eubacterium genus* and *Rumphococcus*). Additionally, there were no other confounding factors and the examined SNPs could only influence constipation through the aforementioned exposure factors, respectively. As a result, the MR results were fairly robust.

**Conclusion:**

Our investigation verified the causal links between the *Eubacterium genus* or *Rumphococcus*, and constipation, with greater *Rumphococcus* expression increasing the likelihood of constipation and the opposite being true for the *Eubacterium genus*.

## 1 Introduction

Constipation is a chronic gastrointestinal disease characterized by a high prevalence. Prolonged constipation symptoms have a detrimental impact on people's standard of life and place a significant financial and social burden on society (Dong et al., 2023). Constipation is also independently linked to poor clinical outcomes like cardiovascular disease (CV disease), end-stage renal disease (ESRD), and mortality, according to recent epidemiological investigations. This association may be mediated by changes in the gut microbiota and increased fecal metabolite production (Dong et al., 2023). Li et al. used Mendelian randomization to analyze the causal relationship between intestinal microorganisms and constipation. The results showed that the abundance of Fecal Coccus increased 1 and the risk of constipation decreased, while the abundance of Pseudomonas increased. Obviously, in the intestinal tract microorganism's fecal coccus genus 1, Pseudomonas and constipation related (Li et al., 2024). The Mancabelli et al. (2017) study found that the abundance of intestinal bacteria, including *fecal* cocci, decreased in patients with constipation. The Yarullina et al. (2020) study found decreased abundance of Fecal Coccus 1 in patients with constipation, but not in traditional probiotics such as *Lactobacillus* and *Bifidobacterium*. The Guo et al. (2020) study on the composition of intestinal microflora in the elderly patients with functional constipation showed that the spectrum of intestinal microflora in the elderly patients with functional constipation was significantly different from that in the healthy population, and the abundance of Bacteroides in the elderly patients with functional constipation was significantly higher than that in the healthy population. Bacteroides are gram-negative anaerobes and play an important role in the degradation and fermentation of colonic organic matter. Wu et al. (2011) confirmed that there was a negative correlation between Bacteroides in feces and dietary fiber intake. Low cellulose diet is the main cause of intestinal flora disorder in constipation patients. Lack of cellulose causes chyme to be difficult to promote intestinal peristalsis and constipation (Xu et al., 2021).

Constipation has also been reported more in Mendelian randomization (MR) studies on cardiovascular disease (CVD; Baumeister et al., 2021). Parkinson's disease (PD; Wang et al., 2023), Growth Factor (GF; Thursby and Juge, 2017) and other factors have been linked to constipation in a direct causative manner. The main treatment for functional defecatory diseases are biofeedback therapy combined with pelvic floor retraining (Bharucha, 2007). Moreover, In the US, constipation is the cause of 2.5 million doctor visits annually, with primary care physicians accounting for more than half of these encounters (Arce et al., 2002). Examining the patient's overall health, mental health, physical ailments, use of dietary fiber, and use of drugs that cause constipation are all crucial. The human gastrointestinal tract has a complex and dynamic microbial population, the intestinal flora, which has a significant impact on the host during homeostasis and disease. These bacteria are kept in a relatively stable state under normal conditions. They play a variety of roles in the body's physiological processes, secrete metabolites that contain enzymes to supplement the body's supply of digestive enzymes, aid in the digestion, absorption, and metabolism of substances, hasten the development of hyperproductive endo-epithelial cells and the regeneration of vascular tissue, maintain the immune system, and stop pathogenic bacteria from proliferating and differentiating (Arce et al., 2002; Cao et al., 2017; Chu et al., 2019). Changes in the composition of gut bacteria are associated with the pathogenesis of many inflammatory diseases and infections (Ohkusa et al., 2019). There is evidence that dysbacteriosis of the gut may lead to functional constipation and constipation-type irritable bowel syndrome. Constipation is made worse when the dynamic equilibrium of the intestinal flora is disrupted, which also affects the body's metabolism and causes a number of pathophysiological changes (Meng et al., 2020). However, studies of gut flora and constipation were not reported. Since there is currently little clinical understanding of constipation, this study looked into the regulation mechanism of intestinal flora in the pathogenesis of constipation as well as a novel approach to treating constipation through intestinal flora management.

Genetic variations are used by MR as instrumental variables (IVs) to determine if a exposure factor has a causal impact on an outcome. The MR offers an alternative way to probe the issue of causality in epidemiological research, by using additional genetic variants that are hypothesized to satisfy the IVs assumptions (Bowden and Holmes, 2019). Meanwhile, MR uses statistical methods from economics to let scientists to examine how human biology and disease are impacted by the environment, medications, and other factors. It is possible to use genetic variants known to influence factors like alcohol consumption or low-density lipoprotein (LDL) levels as IVs that can separate the effects of these factors on outcomes like pregnancy or cardiovascular disease, respectively, by taking advantage of the fact that genetic variation is randomized among children of the same parents. Although MR and similar techniques have limitations that researchers should be aware of, they are becoming more and more effective tools for addressing issues in human biology and epidemiology (Birney, 2022).

Imbalance of intestinal flora leads to a series of clinical symptoms. The disturbance of dynamic balance of intestinal flora leads to the proliferation of pathogenic bacteria, stimulates intestinal mucosal barrier, impairs immune system and leads to the decline of immune function. In addition, the release of a large number of pathogenic bacteria increased human intestinal endotoxin, disrupted the intestinal mucosal barrier, resulting in intestinal dysfunction. *Eubacterium* and *Ruminococcus two* kinds of intestinal microorganism, which can promote intestinal peristalsis, energy metabolism and maintain the balance of intestinal environment. *Eubacterium* is *one* of the *first* discovered metabolic microorganisms. It can degrade cellulose, also can ferment glucose, xylose and so on, and has certain digestive function to resistant starch. Important to Refractory Constipation, an analysis of the composition of the intestinal microflora of fresh feces and the accumulation of feces (old feces) in patients with refractory constipation has found a gap between the two. There are significant differences. The potential causal relationship between constipation and *Eubacterium cocci* and *Ruminococcus coli* was demonstrated.

In this study, based on single Nucleotide Polymorphisms (SNPs) data on constipation and exposure factors (*Eubacterium genus* and *Rumphococcus*) in GWAS database, this research performed Single-Variable MR (SVMR) methods to assess the causal links of *Eubacterium genus* and *Rumphococcus* on constipation separately, and a sensitivity analysis was conducted to assess the impact of hypotheses on the findings and ensure the rationality of findings.

## 2 Methods

### 2.1 Data source and pre-processing

The GWAS ids for constipation (finn-b-K11_CONSTIPATION) originated from the Integrative Epidemiology Unit (IEU) Open GWAS database (https://gwas.mrcieu.ac.uk/). The dataset on constipation contains 16,380,466 single nucleotide polymorphisms (SNPs) from 218,792 samples. The ids of *Eubacterium genus* and *Rumphococcus* were sourced from MiBioGen database (https://gwas.mrcieu.ac.uk/). The sample amount for *Eubacterium genus* (genus Eubacteriumcoprostanoligenesgroup.id.11375) was 17,380 with 656 SNPs. The sample size for *Rumphococcus* (genus.Ruminococcus2.id.11374) was 15339, and the amount of SNPs was 545. Thereafter, the “TwoSampleMR” R package (version 0.5.6; Hemani et al., 2018) was utilized to read and screen SNPs that were strongly linked to exposure factors. The linkage disequilibrium (LD) IVs were then eliminated using *r*^2^ = 0.001 and kb = 10,000 to prevent bias brought on by the chained IVs.

The three main assumptions that underpinned the MR investigation were that: (1) there is a strong and noteworthy association between IVs and exposure; (2) the IVs are independent of confounders; and (3) the IVs can only affect the outcome via exposure and not in any other way.

### 2.2 MR and sensitivity analysis

After the IVs were filtered, the input data for the SVMR analysis were collected. The most MR modes were Inverse Variance Weighted (IVW) regression (Burgess et al., 2015), weighted mode (Hartwig et al., 2017), weighted median (Bowden et al., 2016), simple mode, and MR-Egger (Bowden et al., 2015). Then, odds ratios (ORs) were computed, where a safety factor is represented by a value <1 and a risk factor by a value >1. Three types of plots were created to present the results: scatter, forest, and funnel. Sensitivity analysis, which mostly included the Leave-One-Out (LOO), heterogeneity, and horizontal pleiotropy, was conducted to assess the dependability of the MR data.

## 3 Results

### 3.1 Connection of *Eubacterium genus* and *Rumphococcus* to constipation

**Figure 1:**
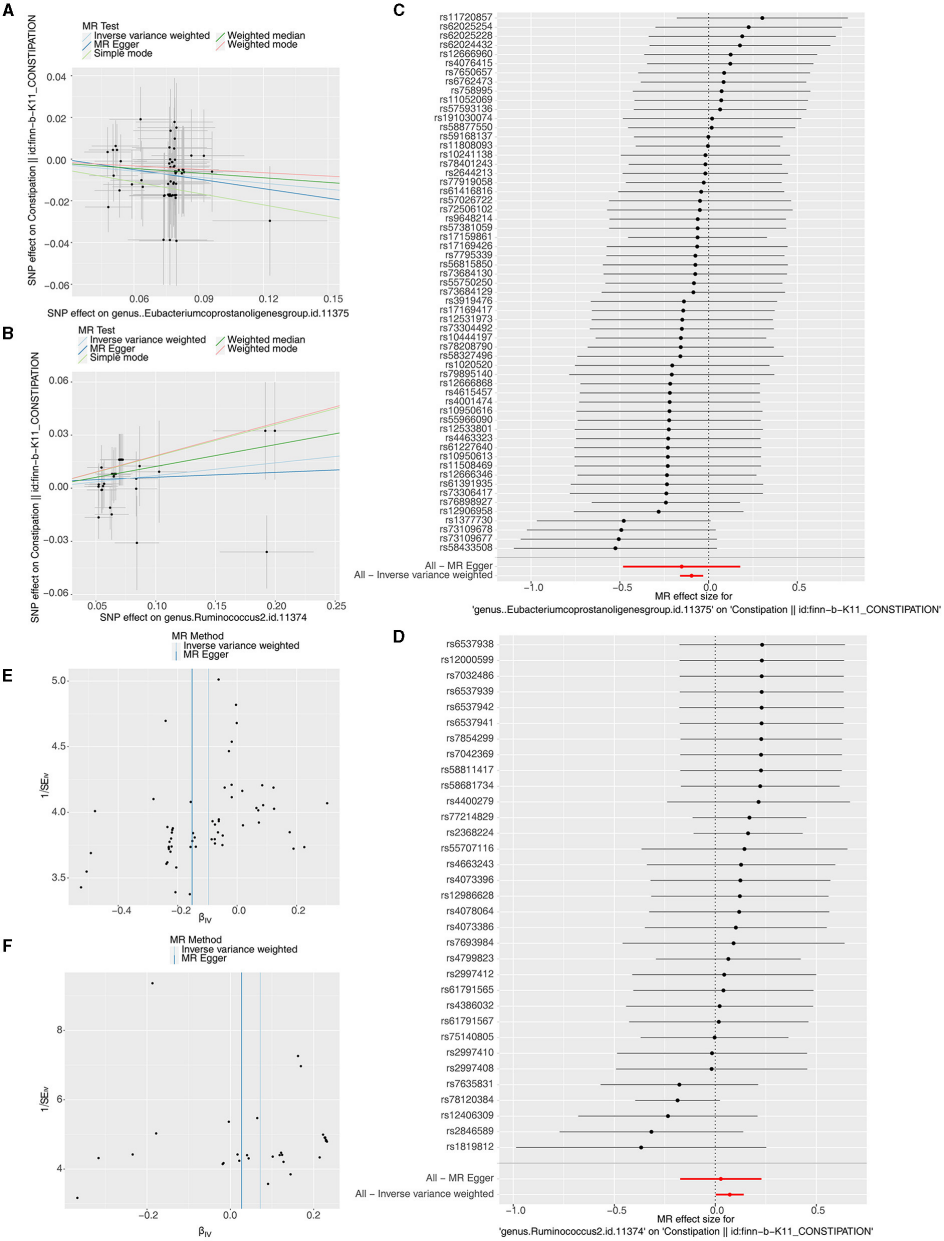
Scatter plots, forest plots, and funnel plots revealing the causative effects of the *Eubacterium genus* and *Rumphococcus* on constipation. **(A, B)** The effects of SNPs on microbiota and constipation. **(A)** *Eubacterium genus*; **(B)** *Rumphococcus*. **(C, D)** MR effect values of microbiota. **(C)** *Eubacterium genus*; **(D)** *Rumphococcus*. **(E, F)** Funnel plots of SNP distributions. **(E)** *Eubacterium genus*; **(F)** *Rumphococcus*.

We identified 66 *Eubacterium genus* and 40 *Rumphococcus* individual SNPs as IVs after screening. The IVW findings suggested that *Eubacterium genus* (*P* = 0.004), *Rumphococcus* (*P* = 0.042) and constipation were linked by causality (Tables 1, 2). After in-depth analysis, OR values revealed that *Rumphococcus* (OR = 1.074) was a risk factor and *Eubacterium genus* (OR = 0.909) was a protective factor for constipation. The scatter plot showed that slope of the line for *Rumphococcus* was positive, indicating that increased amount of *Rumphococcus* led to increased risk of constipation, and the opposite result for *Eubacterium genus* (Figures 1A, B). The SNPs locations in the forest plot were protection on the left and risk on the right. Our findings supported that *Rumphococcus* and *Eubacterium genus* were a risk factor and a protective factor for constipation, respectively (Figures 1C, D). Also, Mendel's second law of random grouping was proven via the funnel plot (Figures 1E, F).

**Table 1:** A test of heterogeneity between *Eubacteriumgenus* or *Rumphococcus* and constipation.

**Id. exposure**	**Id. outcome**	**Outcome**	**Exposure**	**Method**	***Q***	**Q_df**	**Q_pval**	**Id. exposure**	**Id. outcome**
kk6U3A	finn-b-K11_CONSTIPATION	Constipation || id:finn-b-K11_CONSTIPATION	genus..Eubacteriumcoprostanoligenesg roup.id.11375	MR Egger	25.253	57	0.999	kk6U3A	finn-b-K11_CONSTIPATION
kk6U3A	finn-b-K11_CONSTIPATION	Constipation || id:finn-b-K11_CONSTIPATION	genus..Eubacteriumcoprostanoligenesg roup.id.11375	Inverse variance weighted	25.368	58	0.999	kk6U3A	finn-b-K11_CONSTIPATION
moLM5 X	finn-b-K11_CONSTIPATION	Constipation || id:finn-b-K11_CONSTIPATION	genus.Ruminococcus2.id.11374	MR Egger	21.717	31	0.891	moLM5 X	finn-b-K11_CONSTIPATION
moLM5 X	finn-b-K11_CONSTIPATION	Constipation || id:finn-b-K11_CONSTIPATION	genus.Ruminococcus2.id.11374	Inverse variance weighted	21.929	32	0.909	moLM5 X	finn-b-K11_CONSTIPATION

**Table 2:** A test of horizontal pleiotropy between *Eubacteriumgenus* or *Rumphococcus* and constipation.

**Id. exposure**	**Id. outcome**	**Outcome**	**Exposure**	**Egger_intercept**	**SE**	***p*-value**
kk6U3A	finn-b-K11_CONSTIPATION	Constipation || id:finn-b-K11_CONSTIPATION	genus..Eubacteriumcoprostanoligenesgr oup.id.11375	0.004	0.012	0.737
moLM5 X	finn-b-K11_CONSTIPATION	Constipation || id:finn-b-K11_CONSTIPATION	genus.Ruminococcus2.id.11374	0.0039	0.008	0.649

### 3.2 Sensitivity analysis of MR

**Figure 2:**
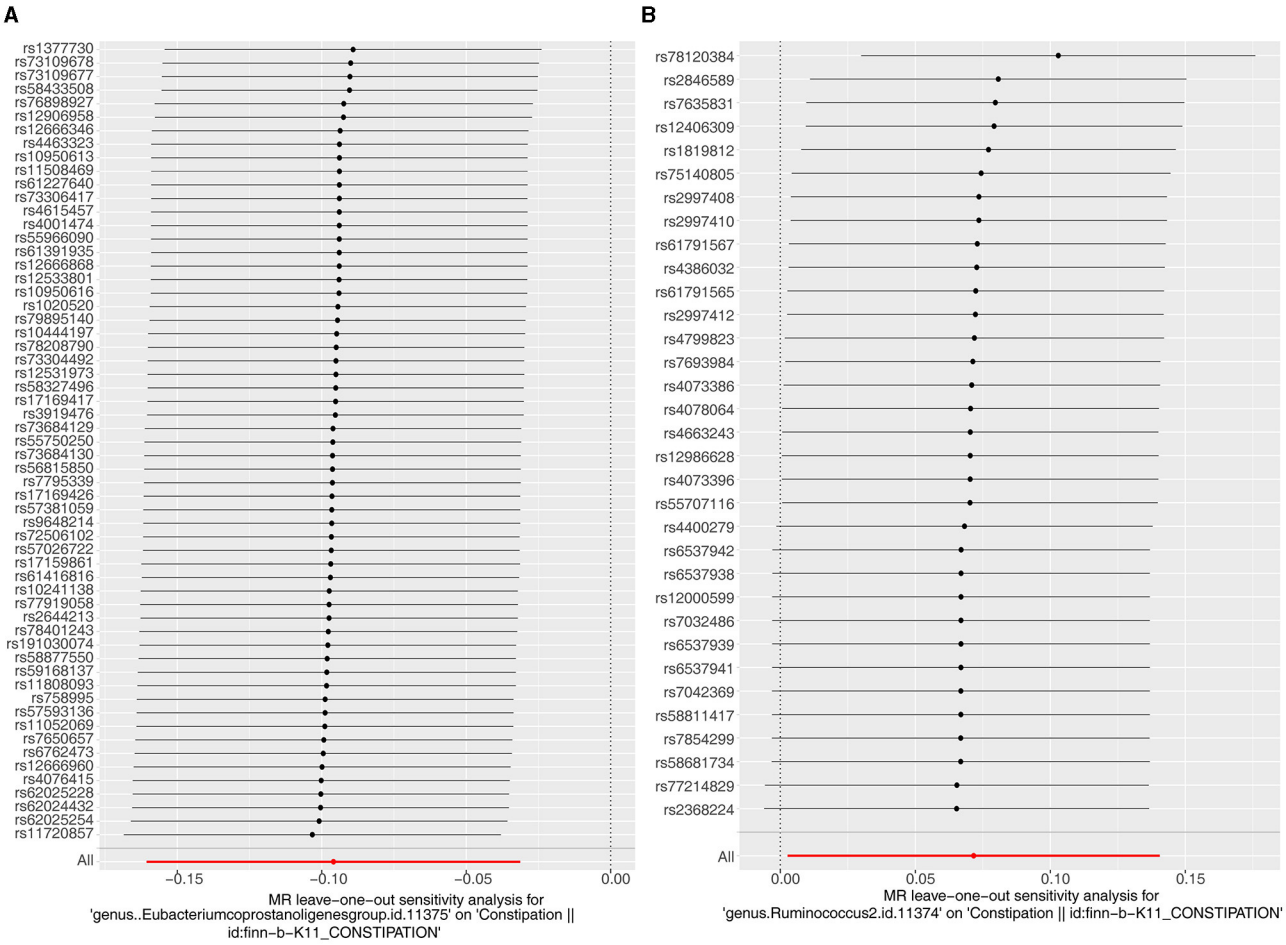
Leave one out of the sensitivity tests. Calculate the MR findings of the remaining IVs after deleting them one by one. **(A)** *Eubacterium genus*; **(B)** *Rumphococcus*.

Several tests were run to assess the dependability of the analyses. The *P*-values of the heterogeneity tests were all larger than 0.05, indicating that there was no heterogeneity between exposure factors and outcomes (Table 1). The outcome of Horizontal pleiotropy test suggested that there was no confounding and SNPs can only influence constipation through two exposure factors independently (*Eubacterium genus*: *P* = 0.737, *Rumphococcus*: *P* = 0.648; Table 2). And, The LOO results reveal that there is no single SNP that has a major effect on the MR results, validating the correctness of the results (Figures 2A, B). In conclusion, *Eubacterium genus* and *Rumphococcus* were causally associated with development of constipation, with *Rumphococcus* as a risk factor and *Eubacterium genus* as a protective factor.

## 4 Discussion

Constipation is one of the most frequent abnormalities of the gastrointestinal system that affects the patient's quality of life (Bisht et al., 2023). Based on SNPs data on constipation and exposure factors in public databases, this work performed a MR analysis using SVMR methods to investigate causality of *Eubacterium genus* and *Rumphococcus* on constipation. And a sensitivity analysis was conducted to assess the durability of the findings. *Eubacterium genus* and *Rumphococcus* were causally associated with constipation, *Eubacterium genus* was a protective factor of constipation, and *Eubacterium genus* was a risk factor for constipation.

It has been documented that each person has their own unique intestinal microbiota profile, which plays a role in nutrient metabolism, maintaining the integrity of the intestinal mucosal barrier, and preventing pathogen invasion. Qualitative and quantitative changes in the gut microbiota are related to the health status of our organism (Black and Ford, 2018). The overall composition of the colonic mucosal microbiota is associated with constipation, and the colonic mucosal microbiota in constipation patients is more abundant in Bacteroides. Probiotics can alter the altered intestinal microbiota of constipation patients, alter intestinal sensory and motor functions, and regulate the intraluminal environment (Di Domenico et al., 2022). Constipation is usually caused by dietary changes or inadequate fiber intake, where treatment with a multi-component probiotic formula consisting of *Bifidobacterium bifidum* and others has a positive effect on relieving constipation symptoms (Zhao and Yu, 2016).

*Eubacterium genus* is one of the first stomach bacteria to be discovered and plays a key role in metabolism. *Eubacterium genus* bacteria obtain nutrients by breaking down cellulose. It is also able to ferment glucose and xylose, which plays a significant role in the digestion of resistant starch. Refractory constipation is the most severe form of constipation, and its cause remains unknown. The symptoms of constipation appear repeatedly, causing great physical and psychological distress to the patient. A growing body of research shows that patients with constipation exhibit significant dysregulation of the gut microbiota compared to healthy individuals (Chen et al., 2021). Constipation is caused by dysbiosis. The study of the gut microbiota can identify the types of bacteria that contribute to the development of constipation (Ohkusa et al., 2019). Someone analyzed the composition of the gut microbiota of fresh feces and accumulated feces (old feces) in patients with refractory constipation and found significant differences between them. This study, we demonstrate a potential causative connection between *Eubacterium genus* and *Rumphococcus* and constipation. *Rumphococcus* is the direct cause of constipation, while *Eubacterium genus* is a protective factor for constipation. This implies that in order to prevent constipation, we should pay close attention to the content of *Rumphococcus* in patients and initiate targeted treatment as soon as feasible. *Ruminococcus* is a human gut symbiont that is found in the baby and adult gut microbiota and has been linked to a variety of intestinal and extra-intestinal illnesses. Meanwhile, *Ruminococcus* is becoming a major player in impacting health and illness outcomes in people ranging from infants to the elderly (Juge, 2023).

Since MR uses IVs analysis to simulate the randomization process of causal reasoning in randomized controlled trials (RCTs), the design is less susceptible to confounding and reverse causal bias, which improves the rigor and persuasiveness of the experiment and makes up for the shortcomings of the existing research design confounding and reverse causal bias, which is the advantage and innovation of this study. However, this study only targets two genera of bacteria, its coverage and generalization are limited, as we all know, constipation is the result of the interaction of a variety of factors, so it is undeniable that there are many other genera that affect the occurrence and development of constipation.

Current conjectures about the metabolic processes in which *Eubacterium* and *Ruminococcus* may be involved, the metabolites produced, and how these products affect the intestinal environment are as follows:

SCFAs are the main metabolites produced by the intestinal microbiota in the colon, such as dietary fiber, through fermentation in the colon (Yumiao et al., 2022). SCFAs are mainly involved in the regulation of intestinal function through the following *two* mechanisms: (1) regulate intestinal function by activating G protein-coupled receptors (GPRs) including GP*R4*1, GP*R4*3, and GP*R1*0*9A* (Li et al., 2022); (2) Regulate the transcription and expression of genes by inhibiting histone deacetylase (HDAC; Li et al., 2022), thereby exerting anti-inflammatory and intestinal barrier functions. SCFAs can maintain the homeostasis of intestinal flora. In a healthy state, the human intestinal microecosystem is always in a dynamic equilibrium. The imbalance of the intestinal microecosystem is closely related to the pathogenesis of the body (Jie et al., 2021), which can lead to low immunity and various inflammatory reactions (Bowen et al., 2020; Shufei et al., 2020). Studies have shown that when the concentration of SCFAs in the intestine increases, H^+^ can be released, thereby reducing the intestinal PH and causing the intestinal environment to form an acidic environment (Li et al., 2022), which can promote the reproduction of probiotics and maintain the homeostasis of the intestinal microbiota. Zichen et al. (2022) found that probiotic compound *preparation*s could increase the content of SCFAs such as acetic acid in mice, promote the proliferation of intestinal probiotics, improve the intestinal microecology of mice, and promote the health of mice. Couto et al. (2020) found that SCFAs can promote the reproduction of probiotics such as Bifidobacteria, which in turn stimulate the synthesis of SCFAs, forming a virtuous cycle, thereby improving the body's intestinal microecology and promoting the balance and stability of the intestinal environment. SCFAs, FC and intestinal microbiota maintain intestinal health and improve constipation symptoms through the complex intestinal microecosystem. Aoki et al. (2016) demonstrated that *Bifidobacterium* gavage in mice could not only reduce loperamide-induced constipation, but also increase fecal SCFAs levels in mice. Matsumoto et al. (2010) also found that probiotic drinks were able to increase the abundance of bifidobacteria and the content of SCFAs, relieving symptoms in patients with constipation. This is consistent with the experimental findings of Aoki et al. (2016). SCFAs affect intestinal motility through the serotonin mechanism: 5-hydroxytryptamine (5-HT), also known as serotonin, produces 95% of 5-HT in the human body from the intestine (Li et al., 2019), which not only stimulates intestinal peristalsis, but is also essential for regulating visceral nerve sensation (Weiping and Bin, 2020). Renying (2022) found that SCFAs in the intestine of mice (B Chinese Journal of Anorectal Diseases, Vol. *4*4, No. *2, 2024, 1*7 acid, butyric acid, etc.) were significantly positively correlated with the level of 5-HT in the colon. Obata and Pachnis (2016) found that SCFAs can increase the level of 5-HT by stimulating ECs, thereby stimulating the vagus nerve and promoting gastrointestinal peristalsis. In addition, animal experiments have shown that *Lactobacillus rhamnosus* strains can specifically increase the concentration of 5-HT and fecal SCFAs in the colon of constipation model mice, thereby enhancing colonic smooth muscle contractility (Wang et al., 2020). SCFAs affect intestinal motility through the choline acetyltransferase mechanism*: C*holine acetyltransferase (ChAT) is an important biological enzyme required for acetylcholine synthesis, and studies have shown that SCFAs can promote the expression of ChAT to regulate neurogenes, thereby affecting intestinal tract movement (Soret et al., 2010). Suply et al. (2012) and Barichello et al. (2015) found that butyric acid in SCFAs, as an energy substrate in colon cells, can increase the proportion of cholinergic neurons and enhance the contraction function of colon muscles by restoring and increasing the content of glial cell-derived neurotrophic factors in mice. Soret et al. (2010) found that butyric acid can reduce the activity of deacetylase (HADC), thereby increasing the ChAT ratio to increase the proportion of cholinergic neurons and promote intestinal motility.

The enteric nervous system (ENS) is the core of the digestive and defense functions of the gastrointestinal tract, and is a complex system of neurons and glial cells in the intestinal wall, including reflex pathways related to normal intestinal peristalsis and sensory function. The gut microbiota is a vast ecosystem of symbiotic bacteria, fungi, viruses and other microorganisms. The gut microbiota not only regulates the motor program of the ENS, but is also essential for the normal structure and function of the ENS. The mechanism by which gut bacteria mediate movement through ENS is unknown. Traditionally, this interaction is thought to occur through immune mediators. However, there is growing evidence to support that the microbiota can interact directly with intestinal neurons. Intestinal neurons have been shown to express TLRs directly, and knockout models have shown a significant reduction in intestinal motility and thus exhibit a constipation phenotype. This suggests that bacteria or bacterial products may interact directly with intestinal neurons to promote ENS maturation, function, and intestinal motility. *5*-HT is a key neurotransmitter in ENS that regulates intestinal motility and secretion responses, and studies have also confirmed that 5-HT can interact with the gut microbiota to initiate secretion and promote intestinal peristalsis by stimulating local intestinal nerve reflexes, and act on the vagus nerve to regulate intestinal contractility (Sikander et al., 2009). A mouse model of antibiotic-induced gut microbiota depletion found that the gut microbiota maintained the integrity of ENS by regulating the activity of intestinal neurons and promoting intestinal nerve development, in which LPS and short-chain fatty acids (SCFAs), the fermentation products of the microbiota, regulated the activity of intestinal neurons, and SCFA stimulated intestinal nerve development, so the gut microbiota is essential for the integrity of ENS (Vicentini et al., 2021). Under the influence of the natural and social environment, diet, brain, intestine and microbiota form a complex two-way network of interactions, which is called the “brain-gut-microbiota axis,” and a large amount of evidence confirms that the “brain-gut-microbiota axis” exists in both directions and plays a key role in the regulation of intestinal motility. Abnormal intestinal microbiota composition may lead to disruption of the brain-gut-microbiota axis signaling pathway, resulting in changes in intestinal motility. Impaired intestinal motility can be the result of dysfunction of control mechanisms at any level from the gut to the central nervous system, or it may be the result of a deficit in central nervous system regulation. The colonization process of intestinal flora initiates the signaling mechanism of the neural circuits that affect motor control and anxious behavior (Heijtz et al., 2011) and therefore plays an important role in the normal development of the central nervous system. It has been found that the gut microbiota is involved in controlling biological responses to stimuli such as digestion, immune system, and mood through the HPA axis (Walker et al., 2013). In addition, probiotics may reduce the expression of the inhibitory neurotransmitter GABA (γ-aminobutyric acid) receptor and the expression of cFos, a marker of neuronal activity, in the brain by modulating the gut-brain axis (Bercik et al., 2011; Bravo et al., 2011; Ait-Belgnaoui et al., 2014); It can also affect the activity of mi RNAs, which may be a mechanism by which intestinal microbes affect brain activity, thereby regulating the expression of important neuronal mRNAs (Laura De et al., 2021). In addition, the gut microbiota has the potential to directly or indirectly affect the nervous system through bacterial cellular components and microbiotic metabolites such as short-chain fatty acids, vita*mins*, and certain neurotransmitters (Badawy, 2017). Studies have shown that probiotics can stimulate intestinal endocrine cells to release gastrointestinal hormones, and immune cells can also stimulate the release of cytokines, which are then transported to the brain through vagus nerve afferents or blood, and the central nervous system changes the function of the gastrointestinal tract through return signals (Kennedy et al., 2017). A recent study found that the liver indirectly senses the intestinal microenvironment, activates hepatic vagus sensory afferents, and transmits signals to the brain, thereby regulating intestinal immune homeostasis through the vagus nerve, including the induction and maintenance of peripheral regulatory T cells (Teratani et al., 2020). Although ENS may be the main regulator of intestinal motility, both ENS and CNS are involved in intestinal motility, and both dysfunction or dysregulation can affect intestinal motility. Studies have shown that probiotics can mediate intestinal motility through the nervous system, providing evidence that probiotics may help regulate the intestinal tract or the central nervous system and normalize intestinal motility. The gastrointestinal microbiota plays a crucial role in intestinal motility, and studies conducted in germ-free mice have shown that both gastric emptying and intestinal motility are prolonged in the absence of gastrointestinal microbiota compared to wild-type mice (Waclawiková et al., 2022). *One* study showed that mice colonized with gastrointestinal microbiota had higher colonic contractility and significantly shorter intestinal peristalsis time (Kashyap et al., 2013). In rats colonized with specific pathogen-free microbiotas, colonization of *Lactobacillus acidophilus, Bifidobacterium* or *Clostridium* tobacco normalized the small intestine migratory motility complex and intestinal transit time, while colonization of E. coli inhibited intestinal electromyoelectric activity. Parthasarathy et al. (2016) found that fecal microbiota composition was correlated with colonic transit time, and the abundance of actinomycetes, bacteroides, lactococcus, and Rossiella was positively correlated with intestinal motility time, while Faecalibacterium was negatively correlated. Diet-induced changes in microbial composition may be partly mediated by changes in intestinal transit time, while dietary effects on intestinal transit time may be partly due to changes in gastrointestinal microbiota function caused by dietary changes. In addition, intestinal bacterial fermentation products can affect intestinal pH, permeability, and increase the production of gases (carbon dioxide, hydrogen, and methane) in the intestinal lumen, thereby reflexively causing intestinal smooth muscle contraction and promoting intestinal peristalsis (Bär et al., 2009).

We will continue to further explore in the future and continuously improve the relationship between microflora and constipation.

## 5 Conclusion

In conclusion, it can be inferred that there is a causal relationship between *Eubacterium genus, Rumphococcus* and constipation in Mendelian studies, respectively, with *Eubacterium genus* as a safety factor and *Rumphococcus* as a risk factor. In other words, an increase in the number of *Rumphococcus* leads to the direct occurrence of constipation, while the opposite is true for *Eubacterium genus*.

## Data availability statement

The original contributions presented in the study are included in the article/Supplementary material, further inquiries can be directed to the corresponding author.

## Author contributions

XZ: Writing – original draft, Writing – review & editing. JC: Funding acquisition, Writing – review & editing. FH: Conceptualization, Writing – review & editing. WD: Project administration, Writing – review & editing. XY: Software, Visualization, Writing – original draft.
